# Knowledge of safe motherhood among women in rural communities in northern Nigeria: implications for maternal mortality reduction

**DOI:** 10.1186/1742-4755-10-57

**Published:** 2013-10-26

**Authors:** Ekechi Okereke, Susan Aradeon, Adekunle Akerele, Mustapha Tanko, Ibrahim Yisa, Benson Obonyo

**Affiliations:** 1 Abt Associates Nigeria, Partnership for Transforming Health Systems Phase 2, (PATHS2), Abuja, Nigeria; 2 Mannion Daniels, Partnership for Transforming Health Systems Phase 2, (PATHS2), Abuja, Nigeria

**Keywords:** Safe motherhood, Maternal mortality, Millennium Development Goals, Northern Nigeria

## Abstract

**Background:**

Most developed countries have made considerable progress in addressing maternal mortality, but it appears that countries with high maternal mortality burdens like Nigeria have made little progress in improving maternal health outcomes despite emphasis by the Millennium Development Goals (MDGs). Knowledge about safe motherhood practices could help reduce pregnancy related health risks. This study examines knowledge of safe motherhood among women in selected rural communities in northern Nigeria.

**Methods:**

This was a cross-sectional study carried out in two states (Kaduna and Kano States) within northern Nigeria. Pretested, interviewer-administered questionnaires were applied by female data collectors to 540 randomly selected women who had recently delivered within the study site. Chi-square tests were used to determine possible association between variables during bivariate analysis. Variables significant in the bivariate analysis were subsequently entered into a multivariate logistic regression analysis. The degree of association was estimated by odds ratio (OR) and 95% confidence interval (CI) between knowledge of maternal danger signs and independent socio-demographic as well as obstetric history variables which indicated significance at p< 0.05.

**Results:**

Over 90% of respondents in both states showed poor knowledge of the benefits of health facility delivery by a skilled birth attendant. More than 80% of respondents in both states displayed poor knowledge of the benefits of ANC visits. More than half of the respondents across both states had poor knowledge of maternal danger signs. According to multivariate regression analysis, ever attending school by a respondent increased the likelihood of knowing maternal danger signs by threefold (OR 2.63, 95% CI: 1.2-5.8) among respondents in Kaduna State. While attendance at ANC visits during most recent pregnancy increased the likelihood of knowing maternal danger signs by twofold among respondents in Kano State (OR 2.05, 95% CI: 1.1-3.9) and threefold among respondents in Kaduna State (OR 3.33, 95% CI: 1.6-7.2).

**Conclusion:**

This study found generally poor knowledge about safe motherhood practices among female respondents within selected rural communities in northern Nigeria. Knowledge of safe pregnancy practices among some women in rural communities is strongly associated with attendance at ANC visits, being employed or acquiring some level of education. Increasing knowledge about safe motherhood practices should translate into safer pregnancy outcomes and subsequently lead to lower maternal mortality across the developing world.

## Background

Developing countries account for about 99% of an estimated half a million maternal deaths that occur each year [1]. A review of the Millennium Development Goals suggests that limited progress is being made to reduce maternal mortality especially across developing countries including Nigeria [2,3]. However interest abounds for community-based approaches to improving maternal health outcomes. One crucial lesson learnt from the Safe Motherhood Initiative is that community involvement is pivotal for sustained reduction of maternal mortality [4]. Community-based interventions can effectively tackle maternal, newborn and child health problems as decisions to seek and access health services are strongly influenced by socio-cultural norms [5].

Nigeria is Africa’s most populous country with a population of over 140 million people [6]. Within the country, there are about 31 million women of childbearing age [7]. Maternal mortality is estimated to be more than twice as high in the rural areas (828 deaths per 100,000 live births) than in the urban areas (351 deaths per 100,000 live births) [7]. Regional variations abound in maternal mortality figures across Nigeria. Evidence suggests that maternal mortality rates (MMR) are significantly higher in northern Nigeria compared to the southern part of the country. The North East and North West zones with MMR of 1,549 deaths per 100,000 live births and 1,025 deaths per 100, 000 live births respectively have rates about ten and six times higher than in the South West (165 deaths per 100,000 live-births) [7,8]. High MMRs in the northern part of the country significantly impacts on the national MMR, estimated at 545 deaths per 100, 000 livebirths [6] which is among the highest in the world [8]. These figures indicate the need for high impact interventions to reduce maternal mortality, while paying particular attention to *rural northern Nigerian communities*.

Ample evidence indicates that when women have greater knowledge through education, there is greater likelihood that they will have better pregnancy and delivery outcomes [9,10]. This is likely because acquiring knowledge through education equips women to make appropriate decisions about their health including during pregnancy and childbirth. A better informed woman is more likely to make appropriate decisions during obstetric emergencies [11], but many developing countries have women with poor education which is more prevalent in rural communities [12] Knowledge and awareness about safe motherhood practices could help reduce pregnancy related health risks [13,14] and promote safer pregnancies and deliveries. Studies suggest that the probability of a woman of reproductive age having more awareness of safe motherhood practices should increase with age, number of deliveries as well as number of antenatal care (ANC) visits by the woman [15]. Two different studies conducted among school students and women in rural Tanzania indicated that there was generally poor knowledge about safe motherhood practices among respondents [13,15]. There is increasing emphasis on the need to provide information to women of reproductive age as a strategy to reduce maternal deaths.

A baseline survey on knowledge of safe motherhood practices was conducted by the Partnership for Transforming Health Systems phase 2 (PATHS2) in two of its supported states (Kaduna and Kano States) in northern Nigeria. This baseline survey was undertaken as a preparatory activity prior to the start of an intervention by PATHS2 – Safe Motherhood Initiative Demand Side (SMID). The aim of SMID is to reduce maternal mortality rates through demand creation for maternal health services and in particular emergency obstetric care services through engagement with community members on the need to access health services especially during pregnancy and childbirth. PATHS2 is a health systems strengthening project sponsored by United Kingdom’s Department for International Development (DfID) and implemented by a consortium of partners led by Abt Associates. PATHS2 works in collaboration with the Government of Nigeria (at Federal and State level) and other stakeholders to improve the planning, financing and delivery of sustainable health services to Nigerians.

This article outlines some key findings from the baseline survey as well as lessons learnt that will support the implementation of the Safe Motherhood intervention across northern Nigeria. It is expected that the SMID intervention will increase knowledge and awareness about safe motherhood as well as help change socio-cultural norms within the intervention communities for improved maternal, newborn and child health outcomes.

## Methods

### Study setting

A cross-sectional survey undertaken in two PATHS2 supported states: Kaduna and Kano States, both situated in the North West geographical zone of Nigeria. Both States have populations that are largely Hausa with some Fulani as the predominant ethnic groups. Kaduna State is the twelfth largest state in Nigeria representing 5% (46,000 sq kms) of Nigeria’s total land mass. The State has an estimated population of 6,094,506 million (according to the 2006 Nigerian national census) spread across 23 Local Government Areas (LGAs). *Kano* State has an estimated population of 9,401,288 according to the 2006 census [16] spread across 44 LGAs and with a total land mass of 20,131 sq kms. Demographic health reports of Kaduna and Kano States indicate a maternal mortality ratio of over 1,000 deaths per 100,000 live births, high total fertility rate of over 7 births per woman and high rates of teenage marriages and child bearing [17,18]. Kano and Kaduna States share a common boundary within northern Nigeria.

Two clusters within these states were selected: Birnin Gwari cluster in Kaduna State and Kunchi cluster in Kano State. Within the PATHS2 cluster system, clusters consist of a group of 13 selected health facilities that provide graded levels of emergency and obstetric care as well as referral services for the purpose of reducing maternal mortality within a population of 500,000 persons. Each cluster is made up of one to four Local Government Areas (LGAs), depending on the LGA’s population size. Birnin Gwari cluster is made up one LGA i.e. Birnin Gwari LGA in Kaduna State while Kunchi cluster spans across three LGAs in Kano State – Kunchi, Tsanyawa and Bichi. Within both of these clusters, rural communities with populations of about 5,000 each or less were selected as SMID communities. For each intervention community, trained community volunteers are expected to transmit useful life-saving health information with the goal of encouraging as well as supporting community members to imbibe safe motherhood practices. This study reports findings from a baseline survey conducted prior to the commencement of the SMID intervention among female community members to assess their level of awareness of safe motherhood practices.

### Sampling and sample size

The sampling frame for the study was the LGAs within the selected clusters in both states. Sampling involved a two stage cluster sampling approach. In the first stage, one cluster was randomly selected among PATHS2-supported clusters within each state prior to the commencement of the SMID intervention. In the second stage, eighteen rural communities were purposively selected from within each cluster as sites for the SMID intervention. In each of the eighteen communities, women who had recently delivered i.e. delivered within the last two years, were selected for the survey and interviewed using structured questionnaires. The minimum sample size required was estimated using Cochran’s sample size formula for categorical data:

n  =  D(Zα)^2^ P(1  ‒  P)/d^2^; Where n is the sample size,

D represents the design effect (taken as 2).

Zα is the standard normal deviate set at 1.96 (for 95% confidence level).

d is the desired degree of accuracy (taken as 0.05).

P is the estimate of the proportion of mothers with knowledge of safe motherhood, taken to be 8.5% [19].

The sample size required was estimated as ~240 per cluster; however factoring a 10% attrition or non-response rate; the estimated sample size per cluster is ~264. Hence 270 persons were sampled from Birnin Gwari cluster (Kaduna State) and 270 from Kunchi cluster (Kano State).

### Data collection

The structured questionnaire for the survey was designed in English but the data collectors were trained using a combination of English and the local Hausa language. The training of data collectors and supervisors lasted for one full day within each state. The questionnaires were translated and back translated for the purpose of training the data collectors/supervisors. The questionnaires were pilot- tested within one of PATHS2’s clusters in Kaduna State and the necessary changes were made to finalize the study questionnaire in preparation for the survey.

The study questionnaire is divided into three sections: *socio*-*demographic characteristics* (which includes questions about age, marital status, ever attended school, education level and occupation of the respondent), *obstetric history* (which includes questions about number of deliveries i.e. parity, number of facility-based deliveries; experience during most recent pregnancy including attendance of ANC and if there were complications during most recent pregnancy), and *knowledge of safe maternal care practices* (which includes questions to evaluate the respondent’s knowledge of the recommended number of antenatal care visits, knowledge of the benefits of ANC visits, knowledge of the diseases/conditions that are prevented by medicines given during ANC visits, knowledge of maternal danger signs, knowledge of necessary actions that pregnant women and their husbands need to prepare for possible maternal emergency and knowledge of the benefits of health facility delivery with a skilled birth attendant). An information sheet and individual consent form were included as part of the format for the structured questionnaire to ensure that the study participants understand and give informed consent to participate in the study.

Community leaders were informed of the research activities prior to the data collection. Taking into consideration the religious and socio-cultural sensitivities within these rural northern communities, only women were selected as data collectors. In each state, there were nine teams of paired *female* data collectors. Local government health officials (Maternal and Child Health [MCH] Coordinators and Health Educators) from the local government health offices also served as supervisors during the data collection. The female data collectors conducted house to house visits in order to apply the study questionnaires based on a randomly selected list of households with women who had recently delivered (i.e. within the past two years). PATHS2 staff from the national office in Abuja as well as the Kano and Kaduna offices provided support during the data collection and supervised the data collection process.

### Data analysis

At the completion of data collection, the data was cleaned, entered into and analyzed using SPSS version 20. For the “knowledge of safe maternal care practices” section of the study questionnaire, the responses of the respondents were assessed against standard safe motherhood guidelines as documented within the Safe Motherhood Initiative Demand Side (SMID) manual developed within PATHS2. Respondents were classified as having “poor” and “good” knowledge based on ability to provide the correct answers to specific questions around safe motherhood as outlined in the structured questionnaire. The minimum correct answer(s) to the safe motherhood knowledge questions for respondent(s) to be classified as having “good” knowledge are outlined in Table 1.

**Table 1:** Minimum correct answer(s) to knowledge questions for respondent to be classified as having 'good’ knowledge

**Question code**	**Safe motherhood knowledge question**	**Minimum correct answer to knowledge question for respondent to be classified as having good knowledge**	**Score attainable**
Q301	Question on the recommended number of antenatal care (ANC) visits	Respondents are expected to know ***the*** correct answer	1
Q302	Question on the benefits of ANC visits for a pregnant woman	Respondents are expected to know at least ***four*** correct answers	4
Q303	Question on the diseases/conditions that are preventable by medicines given during ANC visits	Respondents are expected to know at least ***two*** correct answers	2
Q304	Question on maternal danger signs	Respondents are expected to know at least ***four*** correct answers	4
Q305	Question on necessary actions that pregnant women and their husbands need to prepare for possible maternal emergencies	Respondents are expected to know at least ***five*** correct answers	5
Q306	Question on the benefits of health facility delivery by a skilled birth attendant	Respondents are expected to know at least ***four*** correct answers	4
Q307	Question on necessary clean delivery practices	Respondents are expected to know at least ***four*** correct answers	4
Q308	Question on where the nearest emergency obstetric health facility is to the respondent	Respondents are expected to know ***the*** correct answer	1
**Total correct minimum score attainable for a respondent to be classified as having** '**good**’ **knowledge of safe motherhood practices:**			**25**

For each respondent, obtaining the minimum correct answer(s) to specific knowledge question classifies the respondent as having “good” knowledge for that specific safe motherhood question. To classify a respondent as having 'good’ knowledge of safe motherhood practices, such a respondent should obtain a minimum score of 25 by responding correctly to questions based on specific themes as defined within the safe motherhood guidelines/manual and as outlined within the study questionnaire.

Chi-square tests were used to determine possible association between variables and significance was established when p<0.05. Variables significant in the bivariate analysis were subsequently entered into a multivariate logistic regression analysis. The degree of association was estimated by odds ratio (OR) and 95% confidence interval (CI) between knowledge of maternal danger signs and independent socio-demographic as well as obstetric history variables which indicated significance at p< 0.05. A CI was considered statistically significant when the interval between the upper and lower values did not include one.

### Ethical approval

Research ethical committees in the Ministries of Health in Kaduna and Kano States granted ethical clearance to conduct the study in their respective states. Permission to carry out the study in the selected SMID communities was obtained from local government health officials and community leaders. The aims and benefits of the study, respondents’ right to refuse participation as well as freedom to terminate participation in the study at any time were discussed with each participant. Study respondents were assured of the confidentiality of their identities and responses.

## Results

### Socio-demographic distribution of respondents

The median age of participants in both states and among 540 female respondents was 25 (range: 15 – 49); mean age was 26.9 years. Table 2 shows that more than 50% of women in both states were aged between 21 and 34 years old. Significantly over 50% of respondents in both states indicated that they had attended Qu’ranic schools only as a form of education i.e. informal education, while 13.7% in Kaduna State and 21.1% in Kano State stated that they had completed primary school only as a form of education. Less than 5% of respondents completed secondary school or tertiary education within Birnin Gwari cluster in Kaduna State. About a quarter of respondents in Kaduna State and 15% of respondents in Kano State indicated that there had no form of education. Furthermore over 50% of the study participants in each cluster were in a married polygamous relationship. In addition 55% of female respondents indicated that they were currently employed in Kunchi cluster in Kano State and about 40% of respondents were currently employed in Birnin Gwari cluster in Kaduna State.

**Table 2:** **Socio**-**demographic distribution of female respondents**

		**Birnin Gwari cluster (n=270)**	**Kunchi cluster (n=270)**	**P**-**value**
		**N (%)**	**N (%)**	
**Age of respondents**	< 20 years	57 (21.1%)	71 (26.3%)	0.093
	21-34 years	165 (61.1%)	159 (58.9%)	
	35 - 49 years	48 (17.8%)	40 (14.8%)	
**Ever attended school**	Yes	186 (68.9%)	222 (2.8%)	0.001
	No	84 (29.3	48 (18.2%)	
**Highest Education Completed**	Qu’ranic education only	157 (58.1%)	135 (50.0%)	<0.001
	Primary school	37 (13.7%)	57 (21.1%)	
	Secondary and above	9 (3.3%)	37 (13.7%)	
	None	67 (24.8%)	41 (15.2%)	
**arital Status**	Married(monogamous)	104 (38.5%)	116 (43.0%)	0.402
	Married (Polygamous)	166 (61.5%)	154 (57.0%)	
**Currently employed**	Yes	109 (40.4%)	149 (55.0%)	0.001
	No	161 (56%)	121 (45.0%)	

### Obstetric history of survey participants

One in ten of the female respondents in Kunchi cluster and under ten percent (9.3%) in Birnin Gwari cluster reported being uniparous. Almost half (~45%) of respondents in Birnin Gwari cluster and 36% of survey participants in Kunchi cluster were grand multiparous (parity>5). *But over 60*% *of respondents in each cluster indicated that they had never given birth in a health facility*. A quarter of the survey respondents in Birnin Gwari cluster and almost forty percent of the female respondents in Kunchi cluster reported experiencing a maternal complication during their most recent pregnancy. Significantly, high percentages (65% in Birnin Gwari cluster and 80% in Kunchi cluster) of respondents reported attendance at antenatal care (ANC) visits during their most recent pregnancy (p-value: <0.001). A closer look at Table 3 shows that almost half (~47%) of the survey participants in Birnin Gwari cluster and over half (~53%) of the respondents in Kunchi cluster attended four or more ANC visits during their most recent pregnancy.

**Table 3:** Obstetric history of female respondents

		**Birnin Gwari cluster** (**n**=**270**)	**Kunchi cluster** (**n**=**270**)	**P**-**value**
		**N (%)**	**N (%)**	
**Parity**	Once	25 (9.3%)	31 (11.5%)	0.156
	2 - 5	126 (46.7%)	142 (52.6%)	
	>5	119 (44.1%)	97 (35.9%)	
**Number of deliveries that were facility**-**based deliveries**	Once	60 (22.2%)	56 (20.7%)	0.727
	2 - 5	33 (12.2%)	30 (11.1%)	
	>5	6 (2.2%)	10 (3.7%)	
	Never	171 (63.4%)	174 (64.5%)	
**Experience of maternal complication during most recent pregnancy**/**delivery**	Yes	66 (24.4%)	99 (36.7%)	0.003
	No	204 (75.6%)	171 (63.3%)	
**Attendance at ANC visits during most recent pregnancy**	Yes	176 (65.2%)	217 (80.4%)	<0.001
	No	94 (34.8%)	53 (19.6%)	
**Number of ANC visits to health facility during most recent pregnancy**	No ANC Visits	94 (34.8%)	53 (19.6%)	<0.001
	One ANC Visits	16 (5.9%)	10 (3.7%)	
	Two ANC Visits	16 (5.9%)	24 (8.9%)	
	Three ANCVisits	15 (5.6%)	40 (14.8%)	
	Four or More ANC Visits	129 (47.8%)	143 (53.0%)	

### Source of information about safe motherhood among the female respondents

Table 4 shows that almost 40% in Birnin Gwari cluster and over half of survey participants in Kunchi cluster mentioned that they acquired their information of safe motherhood practices from health talks and discussions in health facilities. While a third (33%) and almost a quarter of the female respondents of the survey in Birnin Gwari and Kunchi clusters respectively indicated that community discussions were the source of their information about safe motherhood practices. About a quarter of respondents in Birnin Gwari cluster and 13% in Kunchi cluster indicated that danger sign songs in the media were the sources of information about safe motherhood. Over 10% of women in Birnin Gwari cluster and almost 30% in Kunchi cluster indicated that friends and neighbours were their sources of information about safe motherhood. However in Birnin Gwari cluster, less than 5% of respondents indicate their spouses, other relatives or danger signs song sung in the community as the source of their information about safe motherhood practices. Similar percentages of survey participants (<5%) report their spouses and danger signs sung within the community as sources of knowledge about safe motherhood within Kunchi cluster in Kano State. Table 5 presents a bivariate analysis between the sources of information about safe motherhood in relation to selected key socio-demographic (age, marital status, ever attended school) and obstetric history (i.e. parity) variables. The percentages among respondents which indicated the different sources of information are presented against these key variables i.e. age, marital status, ever attended school and parity.

**Table 4:** **Source of information about safe motherhood for female respondents** (**n** =**270**)

		**Birnin Gwari cluster (n = 270)**	**Kunchi cluster (n = 270)**	**P**-**value**
		**N (%)**	**N (%)**	
**Community discussions**	Yes	89 (33.0%)	62 (23.0%)	0.012
	No	181 (67.0%)	208 (77.0%)	
**Health talk in a health facility**	Yes	101 (37.4%)	145 (53.7%)	<0.001
	No	169 (62.6%)	125 (46.3%)	
**Danger signs song sung within the community**	Yes	2 (0.7%)	5 (1.9%)	0.247
	No	268 (99.3%)	265 (98.1%)	
**Danger sign song in the media**/**radio**	Yes	66 (24.4%)	35 (13.0%)	0.001
	No	204 (75.6%)	235 (87.0%)	
**Friends and** /**or neighbours**	Yes	37 (13.7%)	80 (29.6%)	<0.001
	No	233 (86.3%)	190 (70.4%)	
**Spouse**	Yes	8 (3.0%)	9 (3.3%)	0.781
	No	262 (97.0%)	261 (96.7%)	
**Other relatives**	Yes	7 (2.6%)	30 (11.1%)	<0.001
	No	263 (97.4%)	240 (88.9%)	

**Table 5:** Source of information about safe motherhood against key characteristics of respondents

	**Health talk in a health facility**			**Community discussions**			**Danger signs song in the media/radio**			**Friends**/**neighbours**			**Other relatives**		
	**Yes (%)**	**No (%)**	**P-value**	**Yes (%)**	**No (%)**	**P-value**	**Yes (%)**	**No (%)**	**P-value**	**Yes (%)**	**No (%)**	**P-value**	**Yes (%)**	**No (%)**	**P-value**
**Age**															
< 20 years	20.0	24.4	0.468	20.0	23.2	0.716	23.8	21.7	0.683	23.9	21.6	0.587	29.7	21.6	0.262
21–34 years	62.4	60.0		63.3	60.1		57.4	62.1		57.3	62.5		48.7	62.2	
35 – 49 years	17.6	15.6		16.7	16.7		18.8	16.2		18.8	15.9		21.6	16.2	
**Marital status**															
Married (monogamous)	42.7	40.9	0.687	38.3	43.0	0.316	45.0	40.8	0.439	40.2	42.1	0.712	47.2	41.2	0.482
Married (polygamous)	57.3	59.1		61.7	57.0		55.0	59.2		59.8	57.9		52.8	58.8	
**Ever attended school**															
Yes	87.8	65.5	<0.001	74.2	76.7	0.533	80.2	74.9	0.267	79.5	74.9	0.304	81.1	75.5	0.445
No	12.2	34.5		25.8	23.3		19.8	25.1		20.5	25.1		18.9	24.5	
**Parity**															
Once	8.6	11.7	0.422	7.9	11.2	0.305	14.9	9.1	0.052	16.4	8.5	0.096	13.5	10.0	0.758
2 – 5	50.2	51.8		52.9	50.1		45.6	52.4		35.3	39.6		43.3	51.7	
>5	41.2	36.5		39.2	38.7		39.5	38.5		0.0	0.0		43.2	38.3	
Never	0.0	0.0		0.0	0.0		0.0	0.0		0.0	0.0		0.0	0.0	

### Knowledge of safe motherhood among the female study participants

Table 6 shows that over 90% of respondents in both clusters displayed poor knowledge of the benefits of health facility delivery by a skilled birth attendant. In addition, over 90% and over three-quarters of respondents in Birnin Gwari and Kunchi clusters respectively displayed poor knowledge of the number of recommended ANC visits. Over 80% of study participants in both clusters displayed poor knowledge about the benefits of ANC visits. Over 50% of respondents in both clusters displayed poor knowledge of diseases preventable by medications given during ANC visits. Furthermore over half and more than three-quarters of the respondents in Kunchi cluster and Birnin Gwari clusters respectively showed poor knowledge of maternal danger signs. All respondents in Birnin Gwari and 97% of respondents in Kunchi cluster had poor knowledge of actions necessary to ensure a safe pregnancy experience. This indicates a clear necessity for women of reproductive age to be informed about and supported in developing as well as implementing a safe pregnancy plan.

**Table 6:** **Knowledge of safe motherhood among female respondents** (n=270)

		**Birnin Gwari cluster (n=270)**	**Kunchi cluster (n=270)**	**P-value**
		**N (%)**	**N (%)**	
**Knowledge of number of recommended ANC visits**	Poor Knowledge	250 (92.6%)	198 (76.2%)	<0.001
	Good Knowledge	20 (7.4%)	62 (23.8%)	
**Knowledge of benefits of ANC visits**	Poor Knowledge	238 (88.1%)	220 (81.5%)	0.034
	Good Knowledge	32 (11.9%)	50 (18.5%)	
**Knowledge of diseases preventable by medications given during ANC visits**	Poor Knowledge	187 (69.3%)	155 (57.4%)	0.006
	Good Knowledge	83 (30.7%)	115 (42.6%)	
**Knowledge of maternal danger signs**	Poor Knowledge	213 (78.9%)	138 (51.1%)	<0.001
	Good Knowledge	57 (21.1%)	132 (48.9%)	
**Knowledge of actions in safe pregnancy plan**	Poor Knowledge	270 (100.0%)	262 (97.0%)	-
	Good Knowledge	0 (0%)	8 (3.0%)	
**Knowledge of benefits of health facility delivery by skilled birth attendants**	Poor Knowledge	255 (94.4%)	243 (90.0%)	0.062
	Good Knowledge	15 (5.6%)	27 (10.0%)	
**Knowledge of clean delivery practices**	Poor Knowledge	236 (87.4%)	198 (73.3%)	<0.001
	Good Knowledge	34 (12.6%)	72 (26.7%)	

During bivariate analysis, Chi-square tests indicated significance between ever attended school and knowledge of maternal danger signs, knowledge of recommended ANC visits and knowledge of diseases preventable by medications given during ANC visits at p<0.05. Being currently employed also showed association with knowledge of maternal danger signs, knowledge of benefits of ANC visits and knowledge of benefits of health facility delivery by skilled birth attendants at p<0.05. In addition, attendance at ANC visits during most recent pregnancy showed significant association with knowledge of maternal danger signs *but parity i*.*e*. *number of deliveries per respondent did not show significant association with knowledge of maternal danger signs at p*<*0*.*05*. Table 7 and Table 8 present the bivariate analysis and multivariate logistic regression analysis on knowledge of safe motherhood among respondents in B/Gwari and Kunchi clusters respectively. These tables indicate the percentage of female respondents that displayed good and poor knowledge for “knowledge of benefits of ANC visits”, “knowledge of maternal danger signs”, and “knowledge of the benefits of delivery by a skilled birth attendant in relation to “ever attended school”, “being currently employed”, “attendance at ANC visits” and “parity”. The p-values, odd ratios and 95% confidence intervals are also presented. The results indicate that when multivariate logistic regression analysis was undertaken, the odds ratio and confidence interval between the association of knowledge of maternal danger signs and ever attended school was (OR 2.63, 95% CI: 1.2-5.8) within Birnin Gwari cluster. Furthermore the odds ratio and confidence interval between the association of knowledge of maternal danger signs and attendance at ANC visits during most recent pregnancy was (OR 3.33, 95% CI: 1.6-7.2) in Birnin Gwari cluster and (OR 2.05, 95% CI: 1.1-3.9) in Kunchi cluster.

**Table 7:** Bivariate and multivariate logistic regression analysis on knowledge of safe motherhood among respondents in B/Gwari cluster/Kaduna State

	**Knowledge of benefits of ANC visits**				**Knowledge of maternal danger signs**				**Knowledge of the benefits of delivery by a skilled birth attendant**			
	**Good knowledge (%)**	**Poor knowledge (%)**	**P-value**	**OR (95%CI)**	**Good knowledge (%)**	**Poor knowledge (%)**	**P-value**	**OR (95% CI)**	**Good knowledge (%)**	**Poor knowledge (%)**	**P-value**	**OR (95% CI)**
**Ever attended school**												
Yes	84.4	68.2	0.061		84.2	66.3	*0.009	2.63 (1.2-5.8)	80.0	69.6	0.392	
No	15.6	31.8			15.8	33.7			20.0	30.4		
**Being currently employed**												
Yes	50.0	39.9	0.027		64.9	34.6	0.047		53.3	40.4	0.023	
No	50.0	60.1			35.1	65.4			46.7	59.6		
**Attendance at ANC visits**												
Yes	60.0	38.2	0.423		84.2	61.5	* 0.017	3.33 (1.6-7.2)	80.0	69.6	0.872	
No	40.0	61.8			15.8	38.5			20.0	30.4		
**Parity**												
Once	33.3	17.8	0.061		8.8	9.6	0.093		12.5	18.7	0.074	
2–5	40.0	64.4			40.4	49.5			62.5	63.0		
>5	26.7	17.8			50.9	40.9			25.0	18.3		
Never	0.0	0.0			0.0	0.0			0.0	0.0		

**Table 8:** Bivariate and multivariate logistic regression analysis on knowledge of safe motherhood among respondents in Kunchi cluster

	**Knowledge of benefits of ANC visits**				**Knowledge of maternal danger signs**				**Knowledge of the benefits of delivery by a skilled birth attendant**			
	**Good knowledge (%)**	**Poor knowledge (%)**	**P-value**	**OR (95%CI)**	**Good knowledge (%)**	**Poor knowledge (%)**	**P-value**	**OR (95% CI)**	**Good knowledge (%)**	**Poor knowledge (%)**	**P-value**	**OR (95% CI)**
**Ever attended school**												
Yes	88.0	81.1	0.249		84.1	79.9	0.035		85.2	81.5	0.636	
No	12.0	18.9			15.9	20.1			14.8	18.5		
**Being currently employed**												
Yes	72.0	51.2	0.027		65.2	45.3	0.039		74.1	52.7	0.044	
No	28.0	48.8			34.8	54.7			25.9	47.3		
**Attendance at ANC visits**												
Yes	81.4	70.2	0.359		86.3	75.4	*0.001	2.05 (1.1-3.9)	57.3	60.6	0.096	
No	18.6	29.8			13.7	24.6			42.7	39.4		
**Parity**												
Once	17.9	20.6	0.083		8.7	14.0	0.097		20.5	26.3	0.089	
2–5	61.4	66.3			55.9	52.2			66.2	59.2		
>5	20.7	13.1			35.4	33.8			13.3	14.5		
Never	0.0	0.0			0.0	0.0			0.0	0.0		

## Discussion

This study set out to investigate knowledge of safe motherhood among recently delivered women in selected rural communities within two States in northern Nigeria, prior to the implementation of a safe motherhood intervention. The findings from this study show that 90% respondents, 80% of respondents as well as over half of respondents in both States displayed poor knowledge of the benefits of health facility delivery with a skilled birth attendant, poor knowledge of benefits of ANC visits and poor knowledge of maternal danger signs respectively. Those that showed good knowledge of safe motherhood practices appear to have obtained their information through discussions with health workers in health facilities, community discussions, from the media, friends, spouses as well as other relatives. A key novelty of this study is that it indicates the sources of information about safe motherhood practices among respondents, unlike most studies undertaken in the past which have a common limitation of not indicating the sources of information about maternal danger signs [15] and safe motherhood practices. With this clearer insight into the sources of information about safe motherhood for respondents, specific interventions can be more appropriately designed and implemented to improve the knowledge of safe motherhood practices especially among women in rural communities.

Discussions with health workers in health facilities (typically during ANC visits) were found to be among the top sources of information about safe motherhood practices for respondents. This is similar to findings elsewhere which suggest that health workers are the key sources of information about safe motherhood for prospective mothers [20]. Community discussions rank second as a key source of information about safe motherhood practices among the study respondents. This strongly emphasizes the need for and the role of community based interventions to ensure that prospective mothers seek adequate health services [21] at emergency obstetric care facilities whenever the need arises. The findings from this study also reiterate the point emphasized by a study undertaken in Gambia [22] that the dissemination of songs about safe motherhood through the media and within rural communities is an effective strategy for delivering useful safe motherhood information. It is however important to systematically disseminate this useful safe motherhood information *within the entire community* (including spouses, relatives, friends and neighbours) and not limit information sharing to prospective mothers.

The study results suggest that the likelihood of knowing the maternal danger signs increases by two or three folds with attendance at ANC visits. In this survey, more than a quarter of the female respondents from both states report that they had experienced some form of maternal complication during their most recent pregnancy or delivery. Some of the key complications indicated by the respondents include severe headache, swelling of the feed/hands/face, convulsions, severe vaginal bleeding, prolonged labour (lasting more than 12 hours), hands/foot/buttocks coming out first, delayed placenta i.e. placenta taking more than 30 minutes to come out as well as severe abdominal pain. The findings from this survey also reveal that about 50% or more of the survey respondents in both states attended four or more ANC visits during their most recent pregnancy, but over 60% of female respondents in both clusters/states indicated that *they had never given birth in a health facility*. Strikingly, this finding corroborates the results of other studies undertaken within northern Nigeria [23,24] in which prospective mothers demonstrate high ANC attendance but record low health facility delivery. A study conducted in Ethiopia suggests that the reasons for not giving birth in a health facility could be due to pregnant women’s lack of knowledge about obstetric danger signs [25] and the impact of these on pregnancy outcomes. It should thus be a priority to ensure that pregnant women attending ANC visits are provided with information about possible obstetric danger signs during pregnancy and delivery as well as the need to seek adequate health care services at emergency obstetric health facilities when necessary.

The study results suggest that being employed increases the likelihood of knowing the maternal danger signs. Better access to information which is associated with being employed could be a plausible explanation [26]. The likelihood of a female respondent having good knowledge about maternal danger signs appear to increase by about three fold if she attends some form of school. This strong association between attending school and knowledge of maternal danger signs is similar to findings in other studies [27-29] and shows that having some form of education significantly increases the awareness of safe motherhood practices. Introducing appropriate health information across all levels of education (including primary and secondary schools) as well as through informal education e.g. Qu’ranic schools for women, will likely improve knowledge of safe motherhood among women of reproductive age.

The implications of the study findings highlight the need for prospective mothers to prepare a safe pregnancy plan as a strategy for improving birth preparedness. Safe pregnancy plans should start with ensuring that prospective mothers have adequate knowledge *and information* about safe motherhood practices while taking into consideration their obstetric history. These safe pregnancy plans should also include specific activities such as setting money aside for maternal emergencies, arranging for a helper to assist a prospective mother, ensuring that there is a driver to assist in cases of maternal emergencies and arranging for blood donors (if necessary) in preparation for a maternal emergency. A key initiative within PATHS2’s SMID intervention is supporting women of reproductive age to develop and implement a safe pregnancy plan to improve pregnancy and delivery outcomes among prospective mothers. In resource poor settings like Nigeria, community based interventions such as the Safe Motherhood Initiative Demand side (SMID) should be given priority resource allocation since safe motherhood service packages increase demand for life-saving health services, reduce maternal mortality and have proven to be cost-effective [30]. The long term impact of safe motherhood interventions is strongly dependent on whether these interventions are carefully designed as well as how sustainability issues are addressed.

This study has three key limitations, first the study was designed as a baseline assessment and communities were *purposively selected* for the Safe Motherhood Initiative Demand Side (SMID) intervention. However selection bias among respondents was minimized by ensuring that the households (where the respondents were identified) were randomly selected. Second, there is the possibility of varying degrees of recall bias as the respondents ranged from women recently delivered within few weeks to women recently delivered two years prior to the study. Third, the study was carried out in *rural* communities and thus the findings from this study are not generalizable to other populations within urban settings across Nigeria. More studies are recommended to determine the relationship between knowledge of safe motherhood and pregnancy experience in *urban settings* across Nigeria as well as in other developing countries.

In conclusion, this study found that there is generally poor knowledge about safe motherhood practices including the knowledge of maternal danger signs among women of reproductive age within rural communities in northern Nigeria. However every woman has the right to make informed decisions about her pregnancy and childbirth. This is more likely where there is equitable access to information about safe motherhood practices. There is a clear need to strengthen and fully utilize the existing sources of information about safe motherhood practices in addition to increasing access to affordable emergency obstetric care services in order to reduce maternal mortality. It is anticipated that the implementation of the SMID intervention will help to address this poor knowledge about safe motherhood which was observed within some rural communities in northern Nigeria. But more importantly, it is hoped that an increased knowledge of safe motherhood practices will translate into safer pregnancy and delivery practices among households in these rural communities.

## Competing interests

All authors declared that they have no competing interests.

## Authors’ contributions

EO and SA jointly designed the study. EO and MT supervised the data collection. EO, AA and MT undertook the data analysis. EO developed the first draft of the manuscript. SA, IY, BO critically revised the draft for substantial intellectual content. All authors read and approved the final manuscript.
