# Correction: Knowledge of safe motherhood among women in rural communities in northern Nigeria: implications for maternal mortality reduction

**DOI:** 10.1186/1742-4755-10-62

**Published:** 2013-12-13

**Authors:** Ekechi Okereke, Susan Aradeon, Adekunle Akerele, Mustapha Tanko, Ibrahim Yisa, Benson Obonyo

**Affiliations:** 1 Abt Associates Nigeria, Partnership for Transforming Health Systems Phase 2, (PATHS2), Abuja, Nigeria; 2 Mannion Daniels, Partnership for Transforming Health Systems Phase 2, (PATHS2), Abuja, Nigeria

## Correction

Following the publication of this work [1], some typographical errors were observed which were not detected during the proofreading process prior to publication. To ensure the complete integrity of the study reported in this publication, corrections in Table two (Table 1 here) are being reflected in this publication of a correction to the article. Sincere apologies are expressed for any inconveniences this error in particular may have caused.

**Table 1:** Socio-demographic distribution of female respondents

		**(n = 270)**	**(n = 270)**	**P-Value**
		**Birnin Gwari Cluster**	**Kunchi Cluster**	
		**N (%)**	**N (%)**	
**Age of respondents**	< 20 years	57 (21.1%)	71 (26.3%)	0.093
	21-34 years	165 (61.1%)	159 (58.9%)	
	35 - 49 years	48 (17.8%)	40 (14.8%)	
**Ever attended school**	Yes	186 (68.9%)	222 (82.2%)	0.001
	No	84 (31.1%)	48 (17.8%)	
**Highest Education Completed**	Qu’ranic education only	157 (58.2%)	135 (50.0%)	<0.001
	Primary school	37 (13.7%)	57 (21.1%)	
	Secondary and above	9 (3.3%)	37 (13.7%)	
	None	67 (24.8%)	41 (15.2%)	
**Marital Status**	Married (monogamous)	104 (38.5%)	116 (43.0%)	0.402
	Married (Polygamous)	166 (61.5%)	154 (57.0%)	
**Currently employed**	Yes	109 (40.4%)	149 (55.2%)	0.001
	No	161 (59.6%)	121 (44.8%)	
